# Shortened Cervix in the Subsequent Pregnancy after Embolization for Postpartum Cervical Hemorrhage

**DOI:** 10.1155/2014/607835

**Published:** 2014-03-30

**Authors:** Zoltan Kozinszky, Sverre Sand, Nils-Einar Kløw, Kirsten Hald

**Affiliations:** ^1^Department of Obstetrics and Gynecology, Blekinge Hospital, Lasarettsvägem 38, 37185 Karlskrona, Sweden; ^2^Department of Obstetrics and Gynecology, Women and Children's Division, Kirkeveien 64 A, 0364 Oslo, Norway; ^3^Department of Radiology, Oslo University Hospital, Ullevaal, Kirkeveien 166, N-0407 Oslo, Norway

## Abstract

*Introduction*. Rupture of a branch of uterine artery during delivery often leads to a massive postpartum hemorrhage that can be successfully treated using uterine artery embolization. *Case Report*. A 33-year-old woman had a cesarean section at term followed by a secondary postpartum hemorrhage due to a ruptured cervicovaginal branch terminating in a large, partially thrombosed hematoma of the cervix. She was given selective uterine artery embolization, and she was discharged to home in stable condition on the third day after embolization. In the forthcoming pregnancy a shortened cervix was a risk of threatened premature delivery from 26 weeks of gestation onwards. *Conclusion*. Superselective unilateral embolization of a thrombosed hematoma in the cervix might prevent extensive iatrogenic trauma of the cervix, which allows preservation of reproductive function.

## 1. Introduction

The rupture of a branch of uterine artery subsequent to a delivery is a rare cause of postpartum hemorrhage that can be often successfully treated by uterine artery embolization (UAE). To our knowledge, no obstetrical followup in the subsequent pregnancy after UAE has been published so far.

## 2. Materials and Methods

A 33-year-old healthy, nonsmoking, normal-weight, primiparous, Caucasian woman had an uncomplicated pregnancy. During spontaneous labor at full term, the cervix was completely dilated after 6 hours, and a relative cephalopelvic disproportion was suspected based on the lack of descent of the fetal head without any clinical sign of chorioamnionitis. A cesarean section was performed via low isthmic incision. Her immediate postpartum course was unremarkable with no pathological bleeding.

**Figure 1:**
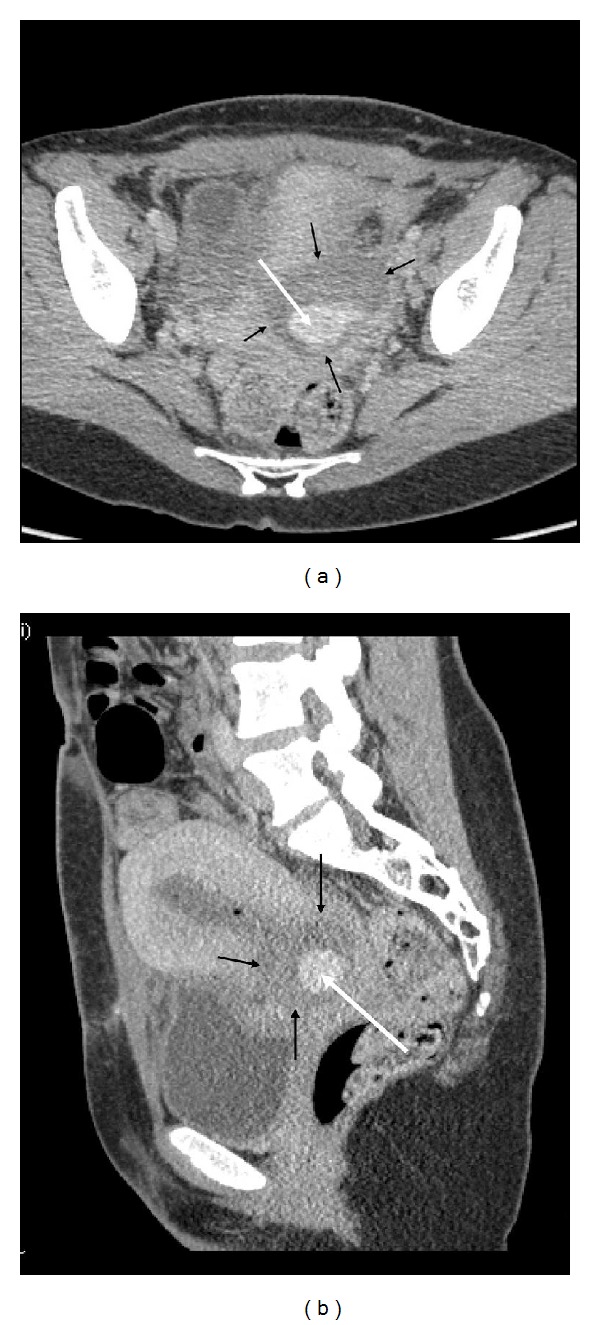
CT angiography of pelvis with axial (a) and sagittal (b) images. The large hematoma is located at the left part of pelvis in the wall of cervix (black arrows). The white contrast shows the leakage into the hematoma (white arrow).

**Figure 2:**
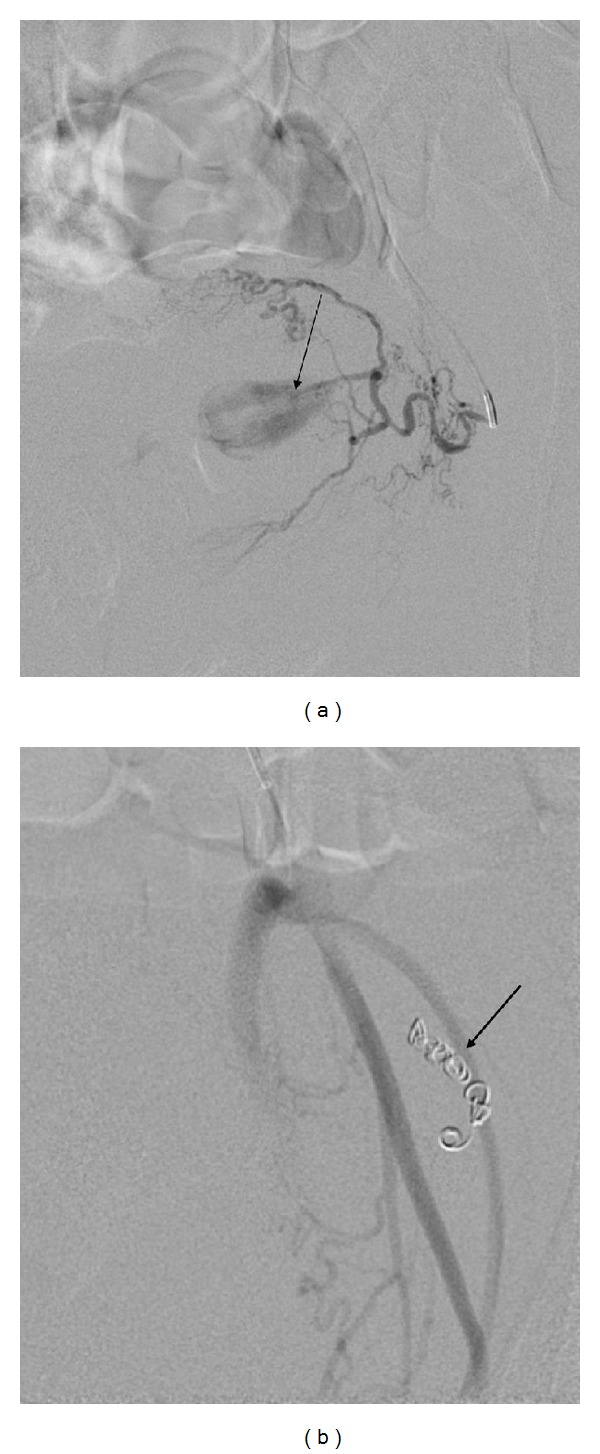
Angiography of the left uterine artery shows contrast leakage from a cervical branch of the artery (a) and complete occlusion of the uterine artery (b) after PVA particles and coils were administered (arrow).

Fifteen days later she was readmitted with persistent uterine bleeding. The cervix was tender by palpation and enlarged with widened external orifice. Doppler ultrasound (US) examination showed an extraluminal collection of blood with turbulent echo within the cervix. Computed tomography (CT) angiography presented a partially thrombosed large hematoma within the wall of cervix (Figure 1). Digital subtraction angiography was carried out on an emergency basis using right-sided transfemoral intervention. Contralateral internal iliac artery angiography revealed contrast media extravasation from a ruptured left cervical arteriolar branch of the uterine artery (UA). CT showed a ruptured branch terminating in a large, partially thrombosed hematoma (6.2 cm × 5 cm × 6 cm) embedded in the left part of the cervix. The thrombus was encircled with the thin wall of the cervix, whereas inside the thrombus, a contrast enhancement was identified representing a hematoma sized 2.5 cm × 2 cm. The cervical arterial branch arose not from the isthmic but from the parametrial part of the left UA, so the bifurcation was distant from the uterine wall in the parametrium forming an uncommon pattern of cervical vasculature. The superselective embolization could not be performed due to spasm of the arteriolar branch. Polyvinyl alcohol (PVA) microparticles (300–500 *μ*m) had been injected to occlude the arteriolar branch and microcoils had been deposited in the distal part of the left UA until complete stasis of antegrade blood flow was achieved [1, 2]. A postembolization arteriogram of the affected branch demonstrated the complete occlusion of the arteriole and that the hemorrhage has not reoccurred (Figure 2). This case report fulfills the criteria of the Declaration of Helsinki.

## 3. Results

The woman became pregnant 15 months following delivery, and early US examination showed a normal cervical length with total resorption of the lesion, whereas at 26 weeks pregnancy the functional cervical length was only 13 mm with a U-shaped funnel (dilation of internal ostium). There were no uterine contractions and no vaginal discharge. Gram staining method manifested normal vaginal bacterial flora. No malformation of the cervix was detected by ultrasound examination. Patient had been hospitalized and on bed rest until an idiopathic premature rupture of the membranes leading to delivery at 36^+2^ weeks of gestation took place.

## 4. Discussion

Bleeding from cervicovaginal branches has been reported previously in some cases [1, 3–5]. However, to our knowledge, this is the first report of a large cervical hematoma due to rupture of a lacerated cervicovaginal branch being the cause of secondary postpartum hemorrhage (SPH). In case of bleeding from the cervical region, selective evaluation and embolization of cervicovaginal branches should be performed in hemodynamically stable patients [1].

Successful embolization for bleeding from a ruptured arteriolar branch/arteriovenous malformation in the cervix has also been described in several reports [1, 4, 5]. We have not found any other report on the obstetric prognosis of embolization of the cervical region. Similarly to hematoma, arteriovenous malformation (AVM) collects embolization material as well. Two cases of cervical AVM have been described till now, but no followup has been presented [4, 5]. One case was successfully treated with UAE [4], whereas in the other case this did not stop the intractable bleeding [5].

The cervix is composed principally of connective tissue (85% extracellular matrix as fibrillar collagen embedded in proteoglycans) and maintains the fetus in situ during pregnancy. The cervical mechanical properties arise from collagen cross-links [6].

Our case report raises the question of previous embolization being the possible cause of weakening of the connective tissue of the cervix. There might be defects in the integrity of collagen matrix in the cervix after embolization treatment. Theoretically, persistent contrast enhancement in the cervical hematoma might produce an irreversible ischemic injury in the surrounding cervical tissues by necrosis and inflammation via damaging of the cervical collagen matrix [6, 7]. Moreover, histopathological studies after embolization have showed local inflammation, edema, and ischemia inducing partial necrosis in the embolized tissue [7].

The defects in extracellular matrix predispose to cervical incompetence in the next pregnancy [6], since a softer cervix cannot resist the intrauterine pressure. Investigation of cervical biopsies from nonpregnant women with a history of cervical insufficiency suggests that increased cervical distensibility during pregnancy can be a result of decreased collagen concentration before pregnancy [6]. Neither other risk for cervical shortening, like vaginal discharge in the current pregnancy, nor uterine infection during previous pregnancy was present in our case. As the short cervix (<15 mm) is the most informative prognosticator of preterm birth independently of obstetrical history [8], we propose a routine serial cervical length assessment during the forthcoming pregnancy after embolization of the pathological condition of the cervix.

Embolization with PVA particles may be associated with more excessive necrosis. Extensive arterial occlusion has been described for more than a year since this agent is used, regardless of particle size [1], whereas Trisacryl Gelatin Microspheres (TGM) allow a more targeted occlusion of blood vessels with a tendency to cause less necrosis depending on the particle size [1]. Perhaps an embolic agent other than PVA, such as resorbable gelatin sponge particles, could have been chosen in order to preserve the intact reproductive function.

In case of cervical pathological feature the embolization should be performed as selectively as possible in order to minimize tissue necrosis; moreover, a definitive revascularization may also be required. Consequently, in our case, the arteriolar spasm by catheter manipulation hampered the superselective procedure, and the use of spasmolytics may have helped to move the catheter further to the bleeding point [2], and theoretically it could have led to a better clinical outcome in the subsequent pregnancy. Further studies on selective versus superselective UAE with PVA versus TGM particles are necessary to evaluate the causality.
